# Cocaine Serves as a Peripheral Interoceptive Conditioned Stimulus for Central Glutamate and Dopamine Release

**DOI:** 10.1371/journal.pone.0002846

**Published:** 2008-08-06

**Authors:** Roy A. Wise, Bin Wang, Zhi-Bing You

**Affiliations:** Behavioral Neuroscience Branch, Intramural Research Program, National Institute on Drug Abuse, National Institutes of Health, Department of Health and Human Services, Baltimore, Maryland, United States of America; University of Granada, Spain

## Abstract

Intravenous injections of cocaine HCl are habit-forming because, among their many actions, they elevate extracellular dopamine levels in the terminal fields of the mesocorticolimbic dopamine system. This action, thought to be very important for cocaine's strong addiction liability, is believed to have very short latency and is assumed to reflect rapid brain entry and pharmacokinetics of the drug. However, while intravenous cocaine HCl has almost immediate effects on behavior and extracellular dopamine levels, recent evidence suggests that its central pharmacological effects are not evident until 10 or more seconds after IV injection. Thus the immediate effects of a given intravenous cocaine injection on extracellular dopamine concentration and behavior appear to occur before there is sufficient time for cocaine to act centrally as a dopamine uptake inhibitor. To explore the contribution of peripheral effects of cocaine to the early activation of the dopamine system, we used brain microdialysis to measure the effects of cocaine methiodide (MI)—a cocaine analogue that does not cross the blood brain barrier—on glutamate (excitatory) input to the dopamine cells. IP injections of cocaine MI were ineffective in cocaine-naïve animals but stimulated ventral tegmental glutamate release in rats previously trained to lever-press for cocaine HCl. This peripherally triggered glutamate input was sufficient to reinstate cocaine-seeking in previously trained animals that had undergone extinction of the habit. These findings offer an explanation for short-latency behavioral responses and immediate dopamine elevations seen following cocaine injections in cocaine-experienced but not cocaine-naïve animals.

## Introduction

Cocaine has multiple pharmacological actions in both the central and peripheral nervous systems. It potentiates the actions of the monoamine neurotransmitters by blocking reuptake through each of the plasmalemmal monoamine transporters [1] and it also acts as a local anesthetic, blocking voltage-gated sodium and calcium channels and calcium-activated potassium channels [2], [3], [4], [5]. The hydrochloride (HCl) salt of cocaine dissociates in solution at physiological pH, and free cocaine readily crosses the blood brain barrier. Its addiction liability derives primarily from its ability to increase dopamine concentration [6], [7] and, thus, dopamine receptor activation [8], [9] in the terminal fields of the mesocorticolimbic dopamine system [10], [11], [12]. Intravenous (IV) cocaine HCl has almost immediate behavioral and cardiovascular effects, effects that have been assumed to result from its rapid entry to the brain [13], [14]. It is thought that the rapid onset of the effects cocaine HCl explains much of its addictive power [15], [16], [17]; it is known that the delay of a single second between an instrumental response and the delivery of reinforcement to the brain can dramatically dilute the effectiveness of the reinforcer [18], [19]. Against the single-second standard, however, the central pharmacological effects of cocaine HCl are not particularly fast. The latency for onset of central pharmacological actions after intravenous injection of cocaine HCl in cocaine-naïve animals appears to be greater than 10 seconds [20] with peak effects tens of seconds later [21], [22]. Meanwhile, cocaine HCl-induced extracellular dopamine increases are evident in cocaine-trained animals at much shorter latency [20], [23]. This very short-latency effect of cocaine HCl is not detected in freely moving animals without prior cocaine experience [20] and it is lost after extinction of the cocaine-rewarded response [24]. A very short latency dopamine response to cocaine HCl can also be seen in cocaine-naïve anesthetized animals [25], but this response is not strong enough to be detected in freely moving animals [20].

Recent electrophysiological and microdialysis studies suggest the possibility that, rather than an effect of blocked dopamine reuptake, the significant immediate dopamine elevation reflects conditioned dopamine *release* triggered by cocaine-predictive cues. Electrophysiological studies show that reward-predictive environmental stimuli come to activate the dopamine system [26]. This activation involves 200 msec bursts of dopaminergic firing triggered by sensory-evoked synaptic input to the dopaminergic cells in the VTA. Microdialysis studies show that cocaine-predictive cues can cause VTA glutamate release, and that this release causes local dendritic release of dopamine [27], a correlate of dopaminergic cell firing [28], [29], [30]. The ability of cocaine to evoke glutamate release in the VTA is seen only in cocaine-experienced, not cocaine-naïve, animals [27], [31].

Among the sensory events that can trigger conditioned responses to ingested substances are the immediate interoceptive cues from the substances themselves [32]. In the present study we used cocaine methiodide (cocaine MI), a quaternary cocaine analogue that does not cross the blood-brain barrier, to determine if the interoceptive stimulus effects of cocaine itself can become conditioned to trigger VTA glutamate release, VTA dopamine release, and cocaine-seeking behavior.

## Results and Discussion

**Figure 1:**
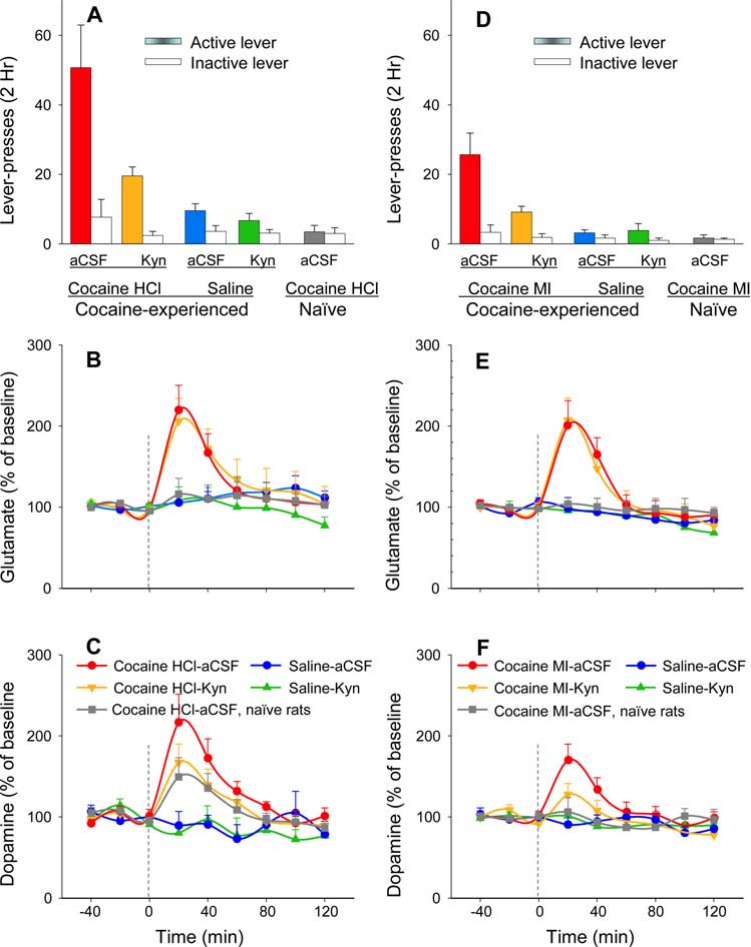
Mean lever-pressing (top panels) and ventral tegmental glutamate (middle panels) and dopamine (lower panels) fluctuations during reinstatement of lever-pressing by cocaine HCl (left panels) and cocaine MI (right panels). IP injections of cocaine HCl (10 mg/kg) or cocaine MI (13 mg/kg) were given at Time = 0. Abbreviations: aCSC (artificial cerebrospinal fluid), Kyn (kynurenic acid, glutamate antagonist), cocaine HCl (cocaine hydrochloride), cocaine MI (cocaine methiodide). The notation “Cocaine HCl-aCSF” indicates the condition where the IP injection was cocaine HCl and where the dialysate included aCSF.

In our initial experiment we monitored ventral tegmental glutamate and dopamine release during cocaine HCl-induced (“cocaine-primed”) reinstatement of cocaine-seeking in an animal model of addiction relapse [33]. Here, rats were first trained to lever-press for intravenous cocaine HCl. After two weeks of daily training, the rats underwent three weeks of daily “extinction” trials in which they were allowed to continue lever-pressing but received only saline injections for doing so. Over the course of this extinction phase, response rates dropped from about 60 responses per hour on the first trial to about 5 presses per hour at the end. (Five presses per hour is about the baseline level of exploratory lever-pressing we see under our testing conditions, the number of lever-presses a naïve and non-rewarded animal makes when first introduced to the test chamber.) In the subsequent reinstatement test, the animals were again tested under extinction conditions but here they were given a 10 mg/kg IP “priming” injection of cocaine HCl just prior to testing. Consistent with earlier studies [34], [35] the IP priming injection caused vigorous resumption of lever-pressing—over 50 responses in 2 h—despite the fact that no further cocaine was earned (Fig. 1 A). The animals responded preferentially on the one of two available response levers that had been associated with injections of cocaine HCl in the training phase (the previously “active lever”), confirming that the response reflected prior conditioning and not a simple increase in general arousal. Minimal lever-pressing was seen in animals given IP priming injections of vehicle (saline). As expected, similar IP injections of cocaine HCl failed to cause significant lever-pressing in cocaine-naïve (saline-trained) animals.

Microdialysis samples were collected from the ventral tegmental area during the challenge session and were assayed for glutamate and dopamine. Glutamate and dopamine levels were each elevated by the cocaine HCl challenge injection in the cocaine-trained rats; dopamine but not glutamate was elevated in the cocaine-naïve (saline-trained) rats (Fig. 1B,C). This is consistent with our earlier finding that unearned IV cocaine HCl causes ventral tegmental glutamate release in cocaine-trained animals but not in saline-trained animals [27], and with the earlier finding that IP cocaine HCl causes ventral tegmental glutamate release in cocaine-experienced animals but not in cocaine-naïve animals [31]. Unsignaled IV cocaine HCl also fails to elevate ventral tegmental glutamate in animals that have had prior experience with unearned cocaine HCl injections—injections that were unpredictable because they were controlled by a “yoked” partner [27]. Thus, with the exception of the present experiment, in all cases where cocaine HCl has been found to cause release of ventral tegmental glutamate, the animals not only had prior experience with cocaine HCl; they had prior experience with cocaine HCl injections that were predictable, either from their own intentional acts or from the repeated ritual of a repeated injection regimen. A possible explanation for the present case—where there was a glutamate response to IP cocaine HCl injections that the animal had *not* experienced previously—is that the peripheral stimulus properties of cocaine HCl, like the external light and sound stimuli in the self-administration experiment [27] and the IP injection ritual in the sensitization experiment [31], served as a predictive cue, a cue that had been previously experienced with IV cocaine HCl injections during the animal's self-administration history. While this possibility may seem unlikely to those not familiar with other studies where the peripheral effects of a drug serve as a conditioned stimulus for their own central effects [32], [36], [37], it was a possibility that we easily tested.

To test the hypothesis that IP cocaine in our first experiment caused glutamate release because its peripheral effects had become conditioned stimuli predictive of cocaine HCl's central effects, we repeated our experiment using IV cocaine HCl as the training drug but using IP cocaine methiodide (MI)—an analogue that has the peripheral effects of cocaine HCl but does not cross the blood brain barrier—as the challenge drug. Like the earlier challenge with IP cocaine HCl, challenge with equimolar IP cocaine MI reinstated drug-seeking and caused ventral tegmental glutamate and dopamine release (Fig. 1D,E,F). Ventral tegmental perfusion with the glutamate antagonist kynurenic acid again attenuated cocaine MI-induced reinstatement of lever-pressing and ventral tegmental dopamine release, confirming that the cocaine MI-activated glutamatergic release was responsible for activation of the dopamine system and for the reinstatement of cocaine-seeking.

While cocaine MI was as effective as cocaine HCl in causing the glutamate release responsible for the initial phase of the dopamine increase (Fig. 1B vs 1E), it was less effective than cocaine HCl at reinstating the cocaine-seeking lever-press habit (Fig. 1A vs 1D) and at causing dendritic dopamine release (Fig. 1C vs 1F). This should not be surprising because cocaine MI provides only the initial glutamate input to the dopamine system, not the sustained blockade of dopamine reuptake that accounts for the bulk of the dopamine elevation seen after self-administered doses of cocaine HCl [6], [7].

We next compared the effects of yoked IV injections of cocaine HCl or cocaine MI on ventral tegmental glutamate and dopamine release in cocaine HCl-trained or saline-trained rats. Here, the injections were paced by the responding of an “executive” animal that earned injections for both itself and its yoked partner. The dosage was 1.0 mg/kg/injection for animals receiving cocaine HCl and 1.3 mg/kg/injection (the molar equivalent of 1.0 mg/kg of cocaine HCl) for animals receiving cocaine MI. The intervals between injections—controlled by the executive partner—were usually 5–6 minutes.

**Figure 2:**
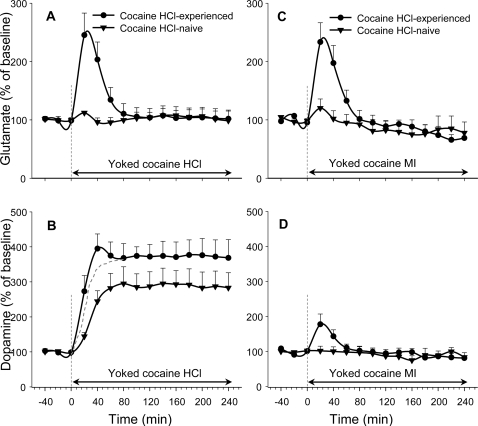
Mean glutamate top panels) and dopamine (bottom panels) fluctuations during yoked injections of cocaine HCl (left panels) or cocaine MI (right panels) in cocaine-naïve animals or animals experienced with cocaine HCl. The dotted line in B represents the effects in cocaine-experienced animals of the peripheral-plus-central actions of cocaine HCl (circles in B) minus the peripheral-only action of cocaine MI (circles in D). The difference between this line and the line reflecting the effects of cocaine HCl in cocaine-naïve animals (triangles in B) is the consequence of sensitization to cocaine in the cocaine-trained animals [40].

Glutamate was elevated equally by the initial yoked injections of cocaine HCl (Fig. 1A) and cocaine MI in cocaine HCl-trained but not in saline-trained animals (Fig. 1B). The elevations were transient; glutamate returned to baseline in the first hour, while cocaine injections continued for another three hours. Dopamine was elevated by both yoked cocaine HCl and yoked cocaine MI, but the elevations were dramatically different (Fig. 2C,D). In cocaine HCl-trained animals, yoked cocaine MI caused a small transient dopamine elevation—an elevation with a similar time-course to that of glutamate. Yoked cocaine HCl, in contrast, elevated dopamine levels in both cocaine HCl-trained animals (Fig. 2B) and in saline-trained animals (Fig. 2D); moreover, it did so for the full 4-hour session (Fig. 2B,D). The elevation was greater in the cocaine HCl-trained animals, consistent with what is seen in cocaine sensitization experiments [38], [39], [40]. The elevation was also more immediate, with an initial peaklet in the cocaine HCl-trained animals; if the response to cocaine MI (Fig. 2D) is subtracted from the response to cocaine HCl (Fig. 2B), the initial peaklet is lost and the dopamine elevations for the cocaine HCl-trained animals simply look a bit larger than those of the saline-trained animals (Fig. 2B, dotted line).

**Figure 3:**
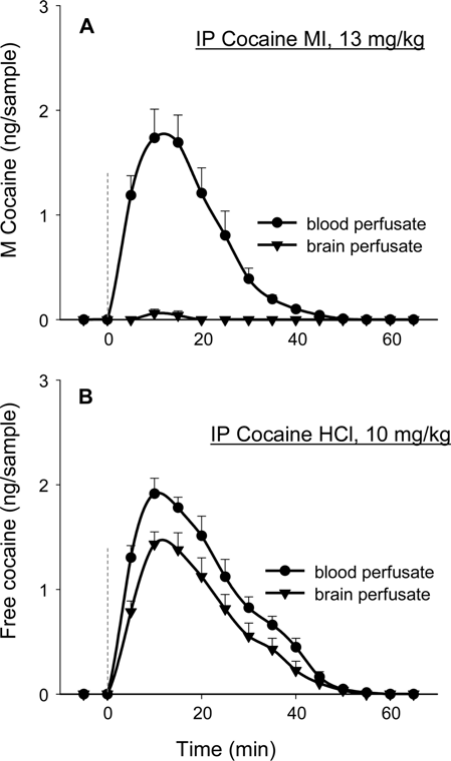
Levels of free and methylated cocaine (M Cocaine) in blood and brain following IP injections of cocaine MI (13 mg/kg) (top panel) or cocaine HCl (10 mg/kg) (bottom panel). Cocaine HCl dissociates at physiological pH and free cocaine enters the brain readily. The methyl group does not dissociate readily from cocaine MI at physiological pH, and the methylated quaternary salt does not cross the blood brain barrier.

**Figure 4:**
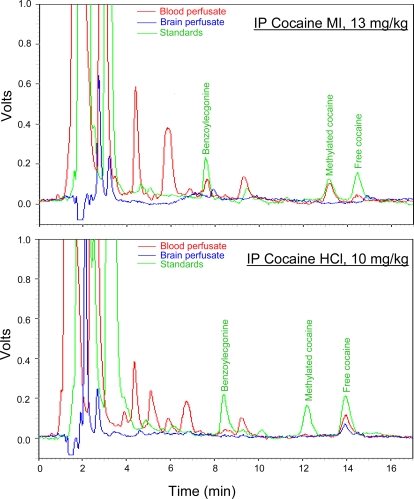
Illustrative chromatograms from blood (red line) and brain (blue line) dialysates taken from an animal given IP cocaine MI (13 mg/kg) (top panel) and an animal given IP cocaine HCl (10 mg/kg) (bottom panel). The green line shows a chromatogram from a standard solution containing free cocaine, methylated cocaine, and the primary cocaine metabolite bonzoylecgonine.

These data are consistent with the view that cocaine HCl has peripheral as well as central actions that contribute the early component to the elevation of extracellular dopamine levels. To confirm that cocaine MI acted peripherally and did not enter the brain, we assayed blood and brain dialysates for cocaine MI (Fig. 3A) and cocaine HCl (Fig. 3B) after IV injections of each. Cocaine HCl was detected in both blood and brain (Fig. 4), with brain concentrations about 25% lower than blood concentration (Fig. 3B; cocaine MI was detected at similar concentration in blood but was not detected in brain (Fig. 3A, 4). These findings offer a simple explanation for the glutamate release seen in cocaine HCl-trained animals that are given so-called “unsignaled” yoked injections of cocaine HCl [27]. While the injections are not signaled by cocaine-predictive exteroceptive cues, the present findings reveal that the yoked injections are signaled by interoceptive cocaine cues, cues associated with the peripheral actions of cocaine.

Our main findings do not, however, explain immediate effects of cocaine HCl that can be detected by fast-scan cyclic voltammetry in cocaine-naïve anesthetized animals [25]. While it is not necessarily the case that conditioned stimulus effectiveness is blocked by anesthesia, the fact that these immediate effects of IV cocaine HCl can be seen in cocaine-naïve animals means that these are unconditioned responses to cocaine HCl. The immediate and unconditioned dopamine response to cocaine HCl in anesthetized cocaine-naïve animals does, however, offer an interesting hypothesis for the development of the conditioned glutamate responses reported here and in our earlier [27] study. First, we must assume that the immediate and unconditioned dopamine response is small and does not reflect the central pharmacological actions of cocaine, as it is not apparent in the dopamine transients seen in awake, freely moving cocaine-naïve animals given IV cocaine HCl [20]. While Mateo et al. interpret their immediate changes in voltammetric signal to reflect a change in dopamine uptake, this is inconsistent with the finding that the central pharmacological effects of cocaine HCl are not evident in awake cocaine-naïve animals for the first 10 seconds after IV injection [20]. We question the assumption that the voltammetric signal reflects a change in dopamine uptake. The finding of Mateo et al. is that IV cocaine HCl increases the dopamine peak elicited by ventral tegmental electrical stimulation, and their impression is that the elevated peak is accompanied by a decrease in the slope of the decay function; this interpretation is based on visual inspection of the decay functions and on a significant increase in apparent K_m_ for those functions [25]. However, the K_m_ estimate is based on the lower portion of the peaks, where the dopamine “signature” of oxidation and reduction currents tends to be lost below the sensitivity threshold for differentiating dopamine from electrical noise [22] and, thus, where the voltammetric signal may no longer reflect dopamine itself. When only the upper portions of the peaks are superimposed, the portions confirmed as dopamine signal, there is no apparent difference in the rate of dopamine clearance (see [25] Fig. 1).

If the effect reported by Mateo et al. reflects a minor and immediate increase in dopamine *release*—rather than a minor immediate change in dopamine *reuptake*—its likely cause could well be a small unconditioned glutamate input to the dopamine system. This brief synaptic input would not be very evident in 20-minute microdialysis samples, but could perhaps correspond to the peaklet seen in Fig. 2A. While this peaklet is statistically insignificant, similar peaklets have appeared in our previous study ([27]: Fig 3B, Cocaine Naïve group and Yoked History group). This small unconditioned glutamate input in cocaine-naive animals might build through Pavlovian conditioning until it becomes the large, obvious, statistically reliable conditioned glutamate input seen in the dialysates of cocaine-trained animals. This is the perhaps the simplest hypothesis of how the conditioned glutamate release arises, an hypothesis that can perhaps be tested with new amperometric methods for rapid glutamate measurements in vivo, methods that have a time scale of seconds [41], [42] rather than the scale of minutes typical of microdialysis studies.

While the present findings were unanticipated and have important implications for addiction theory, they are not inconsistent with current knowledge. It is known that reward-predicting stimuli can activate the dopamine system, and, indeed, can come to substitute for the reward itself in activating the dopamine system [26]. It is also known that environmental stimuli can come to have conditioned behavioral and physiological effects because of their Pavlovian association with addictive drugs, although previous reports have stressed drug-opposite rather than drug-like conditioned effects [43], [44], [45], [46]. It is further known that interoceptive cues—including those associated with drugs [47], [48]—can serve as conditioned stimuli [49]. Finally, it is known that the immediate peripheral effects of a drug can, through Pavlovian association with the later, stronger, central effects of that same drug, play important roles in addiction [32], [36], [37]. Thus the present data simply establish peripheral cocaine discriminative stimulus properties as a subset of the cues that can become associated with the central effects of cocaine and can become conditioned stimuli for cocaine-like effects on extracellular dopamine levels that are mediated by dopamine release rather than by blockade of dopamine uptake.

## Materials and Methods

### Subjects

Fifty-nine male Long-Evans rats (Charles-River, Raleigh, N.C.), weighing 350–400 g at the time of the surgery were used. They were housed individually under a reverse light-dark cycle (light on at 9:30 PM) with free access to food and water. They were allowed to acclimate to the new environment for at least for 7 days prior to surgery. The experimental procedures followed the Principles of Laboratory Animal Care” published by National Institutes of Health (NIH publication 86-23, 1996) and were approved by the Animal Care and Use Committee of the NIDA Intramural Research Program.

#### Intracranial cannulation

Each rat was anaesthetized with pentobarbital (30 mg/kg i.p.) supplemented with chloral hydrate (140 mg/kg, i.p.), and mounted in a stereotaxic frame with the incisor bar 5 mm above the intra-aural line. The skull was then exposed and guide cannulae (CMA/Microdialysis, N. Chelmsford, MA) were implanted above the VTA. Bilateral cannulae were implanted in animals used for the reinstatement studies; unilateral cannulae over the left VTA were implanted in animals used to assay cocaine HCl and cocaine MI in brain. Stereotaxic coordinates for the burr holes were: 5.6 mm posterior to bregma and 2.2 mm lateral to the midline. The guide cannulae were lowered at a 12° angle toward the midline to a depth of 6.8 mm from the skull surface, and cemented with dental acrylic to four stainless steel skull screws.

#### Intravenous catheterization

In the same surgery, a small incision was next made to the right of the midline of the neck and the external jugular vein was externalized and an intravenous silastic catheter (Dow Corning, Midland, MI) was inserted with its tip reaching right atrium. The catheter was secured to the vein with silk suture and the other end was fed subcutaneously around the back of the neck to exit near the back of the skull. The end was slipped over a bent 22-gauge stainless steel cannula (Plastic One Inc., Roanoke, VA) with a threaded head used to secure a dummy cannula and, during testing, a dialysis probe and infusion line. The catheter and the guide cannula were secured to the head pedestal with dental cement and the wound was sutured.

After surgery, each rat was given 0.25 ml of sc 2.27% enrolfloxacine once daily for three days as precaution against infection. The animals were allowed to recover for at least 5 days before the microdialysis or behavioral training started; during this time the catheters were flushed daily with 0.2 mg gentamicin in sterile saline and 0.05 ml heparin (30 U/ml in sterile saline).

Ten of the rats were used in the measurement of cocaine HCl (n = 5) and cocaine MI (n = 5) levels in brain and blood. This was done at the time of surgery, while the animal remained anesthetized. For these animals, guide cannulae were not used; a microdialysis probe (CMA/12 14/02, CMA/Microdialysis) was simply implanted directly into the left VTA to a depth of 8.8 mm from the skull, the same penetration as was used in the behavioral studies. In these animals, a second microdialysis probe (CMA 20 Elite 14/10 PAES, CMA/Microdialysis) was inserted directly into the right jugular vein.

### Cocaine self-administration training

After recovery from surgery, 49 animals were given the opportunity to self-administer IV cocaine in daily (6 days/week) four-hour “training” sessions. No priming or shaping was used. Each rat's catheter was first connected by polyethylene tubing to a microprocessor-controlled syringe pump (Razel Scientific instruments, Stamford, CT), through a feed-through swivel that allowed freedom of movement, and the rat was then placed in a dimly (red) lit operant chamber. The chamber was equipped with two operant levers, one fixed and one retractable (Med Associates, Georgia, VT), 9 mm above the floor. Training sessions began with the insertion of the retractable lever into the chamber and illumination of a 15w house light that remained on throughout the session. Each depression of the retractable lever (designated the “active” lever) caused delivery of intravenous cocaine (1 mg/kg/injection; 6 groups of n = 6–7) or saline (two cocaine-naïve “control” groups; n = 6 each), in a volume of 0.13 ml, delivered over 4.5 seconds. A white cue light above the lever was illuminated with the onset of the infusion and remained illuminated for 20 s during a “time out” in which further lever pressing was ineffective. Depressions of the fixed lever (designated the “inactive” lever) were recorded but had no consequences for the animal. No priming injections or response shaping were given. The training sessions lasted two weeks.

### Extinction training

After the completion of self-administration training, rats to be used in the reinstatement study (two groups, n = 6 and n = 7) underwent extinction sessions. Here each lever-press delivered only saline (0.13 ml/injection) and sessions continued for each animal until its response rate dropped to fewer than 15 responses per 4-h session; this was the baseline level of responding seen in control animals introduced to the chambers with no reinforcement. This criterion was reached after 14–18 sessions with saline substitution.

### Reinstatement testing

Four groups of animals (n = 6–7) were tested for reinstatement of their lever-pressing habit one to four days after extinction trials were completed. Each animal was tested in four conditions, two per day over two days. Two groups were tested with cocaine HCl (10 mg/kg, IP); two were tested with cocaine MI (13 mg/kg, IP). On the first day, half the animals were tested for reinstatement of lever-pressing after an IP challenge injection of their assigned cocaine salt and half were challenged after an IP injection of saline. On the second day the challenges were reversed. For the morning test each day, aCSF was in the dialysate; for the afternoon aCSF plus 1 mM Kyn was in the dialysate. The timeline for this testing is given below.

### Yoked injections

The effects of prior experience self-administering cocaine HCl on the neurochemical effects of cocaine HCl or cocaine MI on ventral tegmental glutamate and dopamine fluctuations were assessed in rats given subsequent unpredictable IV injections of each challenge drug. Two groups (cocaine HCl-experienced; n = 6 each) were trained to self-administer IV cocaine HCl as described above; two groups (cocaine-naïve; n = 6 each) were given the same handling and training routine but with saline as the solution that could be self-administered. One cocaine HCl-experienced group and one cocaine-naïve group were given yoked cocaine HCl; one cocaine HCl-experienced group and one cocaine-naïve group were given yoked cocaine MI.

Yoked cocaine injections are injections that the animal receives passively, under the control of another animal: an “executive” partner to which the experimental animal is said to be “yoked.” The two animals are yoked together in the sense that whenever one animal receives an injection the other animal receives the same injection or a comparison injection at the same time. In the present case, IV injections of cocaine HCl (1 mg/kg/injection: n = 6) or cocaine MI (1.3 mg/kg/injection: n = 6) were passively received by animals yoked to the earned injections of IV cocaine HCl (1 mg/kg/injection) by an executive animal. Here the 4-h sessions were initiated by insertion of the response lever into the cage of the executive animal; response levers were not inserted into the cages of any of the yoked animals, and cue light illumination did not accompany the injections for the yoked animals. Thus the only cues that predicted the yoked injections were the interoceptive cues associated with the injections themselves.

### In vivo microdialysis testing

#### Reinstatement tests

The rats in the reinstatement experiments were connected to the microdialysis systems and placed in the test chambers (with the response lever retracted) the evening before the microdialysis tests. Each rat's blocker was removed from the guide cannulae, the microdialysis probe was inserted and fixed in position, and the probes were connected to the microdialysis pump (CMA/100) with FEP tubing through a three-channel fluid swivel. The probes were perfused over night with an artificial cerebrospinal fluid (aCSF, composition in mM: NaCl, 148; KCl, 2.7; CaCl_2_, 1.2; MgCl_2_, 0.8, pH 7.4) at a flow rate of 0.4 µl/min. The following morning the flow rate was increased to 1.2 µl/min. After 20 minutes for equilibration, a series of five 20-min baseline samples were collected. Then the animals were removed from the chamber, given the assigned challenge injection for that day (one of the cocaine challenges or the saline control challenge), and replaced in the test chamber. The response lever was inserted into the chamber and the animals were allowed to lever-press during two-hour reinstatement tests in which responding was not reinforced. At the end of this test the perfusion solutions were changed to aCSF containing 1 mM of kynurenic acid and the procedure (20 min equilibration, 5 baseline samples, IP injection, testing) was repeated (with the same challenge) for the second session of the day. Comparison groups of untrained (cocaine-naïve) rats were not challenged with Kyn and were tested in a single condition (perfusion with aCSF and challenge with IP cocaine HCl or cocaine MI). The timeline for the trained animals was as follows:

0900h: Kyn in perfusate given to assigned groups
0920h: Baseline sampling begins
1100h: IP cocaine or saline; lever insertion; reinstatement test begins
1300h: End of test, lever retracted.
1500h: Kyn in perfusate given to assigned group
1520h: Baseline sampling again
1700h: IP cocaine or saline; lever insertion; second test begins
1900h: End of test, lever retracted.

#### Yoked injections

Following their training periods, four groups (n = 6 each) underwent microdialysis testing in which they received a series of unearned injections of IV cocaine HCl or cocaine MI injections yoked to the behavior of a rat self-administering an equimolar dose (1.0 mg/kg) of cocaine HCl. Again, the rats were put in the self-administration chambers and connected to the microdialysis systems the night before testing and perfused at 0.4 µl/min overnight. The next morning the infusion rate was increased to 1.0 µl/min and five baseline samples were taken. Then the yoked cocaine MI injections were started. In these tests the response lever was never inserted and the cue light was never illuminated. Samples were collected for four hours.

#### Brain cocaine and cocaine methiodide concentration determinations

Cocaine HCl and MI concentration were determined in blood and brain following IP injection in anesthetized animals. Here three baseline microdialysis samples were collected immediately following insertion of probes into the jugular vein and the VTA. Then the animals were given IP injections of cocaine HCl (10 mg/kg) or cocaine MI (13 mg/kg) and 20-min microdialysis samples were collected for another 80 min. A supplementary dose of anesthetic was given when a rat began to show signs of responsiveness to paw pressure.

### Biochemical analysis of microdialysis samples

#### Glutamate

Glutamate was measured using a high performance liquid chromatography (HPLC) system using a model 582 HPLC pump (ESA, Inc., Chelmsford, MA), a CMA/260 degasser, a CMA/200 refrigerated microsampler, a phase II ODS column (3 µm particle size, 3.2×100 mm, Bioanalytical Systems, Inc., West Lafayette IN), a CMA/280 fluorescence detector and an ESA model 501 data station (ESA, Inc.). The CMA/280 is a fixed wavelength fluorescence detector operating at a maximal excitation of 330–365 nm and emission of 440–530 nm. The precolumn derivatization of glutamate was performed with an *o*-phthalaldehyde/mercaptoethanol reagent (0.4 M borate, 0.04 M phthalaldehyde and 0.4 M 2-mercaptoethanol, pH 10.4). Ten µl of the reagent was added to and mixed with the samples by the microsampler. After a 60 s reaction period at 6°^lo^C in the microsampler, 20 µl of the mixture was injected onto the column. The elution of glutamate was achieved with a mobile phase consisting of 0.15 M sodium acetate, 10% methanol and 1.5% tetrahydrofuran at a flow rate of 0.6 ml/min. Following the appearance of the glutamate peak on the chromatogram, an injection of 20 µl of 100% methanol was made by the microsampler before the end of the chromatogram in order to accelerate the elution of the residuals on the column. The detection limit was 0.2 pmol/injection.

#### Dopamine

Dopamine was measured using a HPLC system using an ESA Coulochem II Detector (model 5200) with a dual-electrode microdialysis cell, and an ESA model 501 data station (ESA, Inc.). Samples were manually injected onto the column (3 µm particle size, 3 mm×150 mm, Analytical MD-150, ESA, Inc.). The mobile phase for DA separation consisted of 75 mM NaH_2_PO_4_, 1.5 mM OSA, 10 µM EDTA, and 8% acetonitrile (pH 3.0 adjusted with H_3_PO_4_) delivered at a flow rate of 0.5 ml/min. Dopamine was quantified on both reducing (−250 mV) and oxidizing electrodes (350 mV). The limit of detection for dopamine was approximately 5-fmole/injection.

#### Cocaine HCl and cocaine MI

Cocaine HCl dissociates in saline solution at physiological pH, and free cocaine elutes early under our HPLC conditions. The methyl group in cocaine MI does not dissociate from free cocaine in saline solution at physiological pH, and methylated cocaine elutes much later than free cocaine under our conditions. Thus we looked for cocaine and methylated cocaine peaks in brain and blood samples of animals given IP injections of cocaine HCl (10 mg/kg) or cocaine MI (13 mg/kg) samples. The mobile phase consisted of 0.25 M sodium acetate, 0.1 mM tetrabutylammonium phosphate, 15% methanol and 10% acetonitrile. The mobile phase was adjusted at pH 6.5 for optimal separation of the two drugs and delivered onto the column (5 µm particle size, 2 mm×250 mm, Capcell-C18, ESA, Inc.) at a flow rate of 0.4 ml/min. Cocaine and methylated cocaine were detected using a Waters 2475 Multi λ Fluorescence Detector (Waters Corp., Milford, MA) with excitation and emission wavelengths set at 225 and 315 respectively and quantified by the ESA data system. The detection limits for cocaine and methylated cocaine were approximately 0.1 ng/injection.

### Drugs

Cocaine HCl, cocaine MI, and the anesthetic used in the surgery were obtained from the pharmacy department within the institute. Kyn and chemicals used for HPLC were purchased from Sigma-Aldrich Corporation (St. Louis, MO). Kyn was first dissolved with a small aliquot of 5N NaOH and diluted with aCSF to the final concentrations. The drug solution was adjusted to pH 7.4 before use. Cocaine HCl and cocaine MI were dissolved in physiological saline at equal molar concentration for both IP injection and intravenous infusion.

### Histology

After the completion of the microdialysis experiments, the rats were decapitated under anesthesia and the brains were removed and post-fixed for 7 days in 10% formalin solution. The brains were then frozen and 50 µm coronal sections were taken. Probe placement was determined under low magnification in wet sections that differentiate fiber bundles from cell body regions and thus identify classic brain landmarks.

### Statistical analysis

The substance levels are expressed as the concentrations found in the perfusate (means±S.E.M.). Basal values refer to those obtained before the drug was infused or before the start of behavioral testing. When data are expressed as percent of baseline values, the mean concentration of the three samples preceding the drug infusion or the behavioral test is defined as 100%. The drug effects between treatment groups were analyzed with two-way analysis of variance (ANOVA) with repeated measures over time followed by Fisher's PLSD test. The drug effect within a treatment group was analyzed with one-way ANOVA with repeated measures over time followed by Dunnet's test. The effects of the drugs on lever-press rate were analyzed using two-way ANOVA with repeated measure over time followed by Fisher's PLSD test. The level of *P*<0.05 was the criterion for statistical significance
